# Time Perspective, Psychological Well-Being and Attitudes to Seeking Mental Health Services in Russian Y and Z Generations

**DOI:** 10.3390/ejihpe15050067

**Published:** 2025-04-29

**Authors:** Evgeniy B. Bashkin, Galina N. Kameneva, Vsevolod Konstantinov, Irina A. Novikova, Tatiana S. Pilishvili, Marina A. Rushina, Dmitriy A. Shlyakhta

**Affiliations:** 1 Psychology and Pedagogy Department, Peoples’ Friendship University of Russia Named After Patrice Lumumba (RUDN University), 117198 Moscow, Russia; bashkin_eb@pfur.ru (E.B.B.); kameneva_gn@pfur.ru (G.N.K.); pilishvili_ts@pfur.ru (T.S.P.); rushina_ma@pfur.ru (M.A.R.); shlyakhta_da@pfur.ru (D.A.S.); 2 Department of General Psychology, Penza State University, 440026 Penza, Russia

**Keywords:** attitudes toward seeking mental health services, psychological help, time perspective, psychological well-being, Y and Z generations

## Abstract

Help-seeking of mental health professionals remains low, even in countries where mental health care is highly accessible. Therefore, it is necessary to continue research on the sociodemographic and psychological factors of attitudes toward seeking mental health services (ATSMHS) in different countries. The purpose of the present study was to identify the associations of the ATSMHS with psychological well-being and time perspective in the Russian Y and Z generations. The Y-Generation sample included 217 (69.9% female) respondents aged 22 to 39 years, and the Z-Generation sample included 256 (82.8% female) respondents aged 17 to 21 years. Russian versions of the Psychological Well-Being Scale (PWBS) by Ryff, Zimbardo Time Perspective Inventory (ZTPI), and Inventory of Attitudes toward Seeking Mental Health Services (IASMHS) by Mackenzie and colleagues were used for diagnostics. The main research findings show that (1) Personal growth is a positive predictor and the Fatalistic present is a negative predictor of ATSMHS in both generations, and (2) additional positive predictors of ATSMHS in the Y–Generation are Positive relations and Hedonistic present, and in the Z–Generation it is Future. These findings are important for developing programs to maintain, preserve, and prevent mental health across generations.

## 1. Introduction

### 1.1. Mental Health and Help-Seeking Attitudes

The World Health Organization (WHO) defines mental health “as a state of mental well-being that enables people to cope with the stresses of life, realize their abilities, learn well and work well, and contribute to their community”. It has intrinsic and instrumental value and is integral to our well-being” (World Health Organization, 2025).

The WHO European Declaration on Mental Health recognizes the importance of examining mental health care-seeking behavior as a public health priority. Despite the widespread prevalence of mental health problems among various population groups in different countries, leading to a decrease in quality of life and well-being, help-seeking behavior in the field of mental health remains low, even in countries where psychological and psychiatric care (mental health care) is highly accessible (Antonova, 2020). The majority of individuals with mental health problems remain untreated because they prefer antidepressant medication use when seeking treatment for mental health problems (Mackenzie et al., 2014), and many people who have suffered trauma refuse or hide from seeking professional help (Kantor et al., 2017).

The attitude toward seeking professional psychological help as an empirical psychological concept was proposed about half a century ago by Fisher and Turner (Fisher & Turner, 1970). The authors attempted to determine the tendency of people experiencing a personal crisis or long-term psychological distress to seek psychological help or resist the possibility of receiving it.

To study the psychological factors that promote and hinder seeking professional psychological help, Fisher and Turner developed the Attitudes Toward Seeking Professional Psychological Help Scale, ATSPPHS (Fisher & Turner, 1970). When developing the technique, the authors identified four factors of attitudes toward psychological help: (1) recognition of one’s own need for professional psychological help, (2) tolerance of stigma associated with psychiatric help, (3) interpersonal openness regarding one’s own problems, and (4) trust in a mental health professional. The limitation of this 29-item technique’s version is that it identifies the attitudes, first of all, of students as subjects exclusively toward psychologists and psychiatrists, without touching upon other specialists who have appeared in connection with the development of theoretical concepts and the helping professions industry. Later, Fischer and Farina developed the 10-item *ATSPPH short form* (ATSPPH-SF), which includes three distinct dimensions: (1) openness to seeking professional help, (2) value in seeking professional help, and (3) preference to cope on one’s own (Fischer & Farina, 1995). Both forms of the ATSPPHS are used for research in different languages in their adapted and modified versions.

Mackenzie et al. modified the ATSPPHS and developed the *Inventory of Attitudes toward Seeking Mental Health Services* (IASMSH) to overcome the existing limitations of the ATSPPHS. The IASMSH consists of 24 items combined into three scales: (1) Psychological Openness, reflecting the tendency to recognize the existence of psychological problems and possibilities for their professional solution; (2) Help-seeking Property, reflecting the desire and ability to seek professional psychological help; and (3) Indifference to Stigma, reflecting concern about the opinions of others about a person who has sought psychological help (Mackenzie et al., 2014). The IASMHS has been adapted and validated in different countries (Austria, France, Ireland, Philippines, Portugal, Russia, etc.), and its three-factor structure has been confirmed in most versions (Brás et al., 2022).

Today, access to mental health services continues to present challenges for various populations. Despite the increasing amount of social media information about the possibility of seeking various mental health services, it may increase both the risks and opportunities for help-seeking users in different age groups (Godleski et al., 2024).

### 1.2. Factors and Predictors of Psychological Help-Seeking Attitudes

Among the circumstances under which subjects refused treatment were attitudinal barriers (Lemmer et al., 2024), treatment prices (Yasmin et al., 2024), the need for expensive insurance documents, and stigma-related concerns (Z.-X. Chen & Chandrasekara, 2016; Choi et al., 2024) self-stigmatization (Z.-X. Chen & Chandrasekara, 2016; Yasmin et al., 2024). Attitudes toward help-seeking and stigma were the leading predictors of help-seeking; however, this did not determine the refusal to receive professional help. At the same time, the ability to receive social support significantly determined the refusal of nonprofessional help (Yakunina et al., 2010). A tendency to have a negative attitude toward seeking support was found even among medical students who were indifferent to the issue of stigma (Amin et al., 2023).

Using different methodologies and inventories, numerous studies have been conducted on factors and predictors that may affect psychological help-seeking attitudes and their constituents, in particular, *sociodemographic, socio-cultural*, and *psychological* factors.

Picco et al. (2016) study found significant associations between the ATSPPH-SF factors and *sociodemographic* variables, including age, ethnicity, marital status, education, and income. According to the authors, subgroups of the population who are less open or see no value in seeking psychological help should be reached through culturally sensitive educational programs and supportive individual supportive work (Picco et al., 2016).

Among sociodemographic factors, gender and age are the most studied. In particular, female students compared to male students have more positive attitudes, intentions, and actual help-seeking behavior (Z.-X. Chen & Chandrasekara, 2016; Rickwood et al., 2005). Male ideals of masculinity are significantly associated with the avoidance of seeking psychological help (Yousaf et al., 2015).

Russian research also shows that men have more negative attitudes toward professional psychological help than women and have lower levels of psychological literacy (Anikina et al., 2020). Do nothing and self-care when seeking help were revealed among male students as having a low mental health literacy level (DeBate et al., 2022). Gender differences were found in the attitudes of the participants, with more pronounced indicators of help-seeking in the female sample (Hamid et al., 2009). Age differences are shown in the more frequent professional help-seeking by older people when their health deteriorates, compared to young people (Ropret et al., 2023). Some studies have found that older adults have more positive attitudes toward help-seeking and lower stigma than younger adults (Gonzalez et al., 2005; Mackenzie et al., 2019).

A number of studies have been conducted in different countries, which have shown the influence of *socio-cultural* and *ethno-cultural* factors. Canadian Muslim participants chose to seek help from peer groups, family, and community rather than seeking professional help (Belal et al., 2022). The interaction between gender, age, ethnicity, and educational indicators in receiving general and specialized medical care was studied (Gonzalez et al., 2011). For African Americans, mental health care effectiveness perception has been improved compared to non-Latino whites (Gonzalez et al., 2011).

A study conducted among Arab students confirmed that higher rates of seeking professional psychological help were among medical students, female students, students with positive counseling experiences, and low stigma (Rayan & Jaradat, 2016). Torres et al. (2021) aimed to identify differences in the ATSPPH-SF scores scale across subgroups of Latino adults by gender, country of origin, and English or Spanish language status. Among Chinese participants, an overall negative score on the ATSPPH-SF, a neutral attitude on the openness scale, and a negative attitude toward the value and necessity of help-seeking were found (P. Chen et al., 2020). Interestingly, in a cross-cultural comparison of students from China and Germany, higher scores were obtained among the Chinese on the openness to help-seeking scale, even taking into account the long-standing and traditional psychotherapeutic help in Germany (Zhou et al., 2019).

Most *psychological* research on mental health help-seeking has focused on stigma and the assessment of mental health literacy (Z.-X. Chen & Chandrasekara, 2016; DeBate et al., 2022; Foster & O’Mealey, 2022; Mackenzie et al., 2019; Őri et al., 2023; Rayan & Jaradat, 2016; Shahwan et al., 2020; Swisher et al., 2024; Vogel et al., 2013; Yasmin et al., 2024; Zhou et al., 2019). Swisher et al. (2024) found that more positive attitudes toward seeking help were associated with lower self-stigma, public stigma, and having received psychological treatment in the lifetime of Hungarian respondents. Shahwan et al. (2020) demonstrated that destigmatization interventions three months after their implementation showed a significant increase in help-seeking attitudes among students. Sharp et al. (2006) also showed that detailed information about mental health services was associated with students’ more positive attitudes toward seeking help.

Among other psychological correlates and predictors of attitudes toward seeking mental health services (ATSMHS), time perspective and psychological well-being are often studied (frequently in conjunction with sociodemographic and socio-cultural variables).

#### 1.2.1. Time Perspective

Gonzales and Zimbardo (1985) conducted a time perspective investigation as a stable framework for the existence of a person’s mental life on a sample of more than 12,000 respondents from different countries (Gonzales & Zimbardo, 1985) The authors identified five factors: negative past, hedonistic present, future, positive past, and fatalistic present, which were included as subscales in the Zimbardo Time Perspective Inventory, ZTPI (Zimbardo & Boyd, 1999). ZTPI was adapted in many countries, including Russia. The Russian version of the ZTPI almost completely confirmed the factor structures in the Russian-speaking sample (Sircova et al., 2008).

The authors of previous studies on time perspective as an ATSMHS predictor are based on the fact that help-seeking is a decision-making process, and almost every decision-making is affected by individual time perspectives in accordance with the ideas of Zimbardo (Gonzales & Zimbardo, 1985; Zimbardo & Boyd, 1999). Thus, Kiss et al. (2020), in a theoretical meta-analysis, identified possible drivers for seeking professional psychological help based on a number of previous studies: (a) past negative time perspective through perceived suffering pressure motivation; (b) future time perspective through motivation to prevention; (c) female gender through openness; (d) living in the capital; and (e) previous help-seeking experience. Barriers to seeking professional psychological help include: (a) perceived stigma through anticipated social exclusion; (b) present fatalism through helplessness and external control; (c) male gender through rigid masculinity; (d) living in the countryside; and (e) cost. At the same time, these authors, in their empirical study on a Hungarian sample, found that ATSMHS was positively associated with female gender and inversely associated with stigma and living outside the capital, but none of the time perspective scales were associated with ATSMHS (Kiss et al., 2020).

Canadian scientists in Winnipeg examined the relationship between time perspective and ATSMHS in two age groups among young (18–25-year-olds) and older participants (60–89-year-olds) as they read a health-focused brochure, taking into account their future and present orientations. The authors found that manipulating time perspective did not affect recall of information in the brochure or changes in help-seeking intentions, but the relationship between time perspective and liking the brochure was significant. It has been shown that older participants who are more focused on the present are more motivated to seek help attractively, which needs to be taken into account to improve the psychological literacy of older people (Belal et al., 2022).

Research on mental health help-seeking among university students in Malaysia has shown that Chinese ethnicity, long-term orientation, Hindu religion, masculinity orientation, psychological well-being, Malay ethnicity, and participants’ age were significantly associated with attitudes toward mental health help-seeking (Koon et al., 2023). Positive past experiences, emotional competence, health literacy, and social environment contribute to help-seeking behavior (Rickwood et al., 2005).

#### 1.2.2. Psychological Well-Being

The concept of Psychological well-being was developed by Ryff (1989) as a basic subjective construct reflecting the perception and functioning assessment at the peak of human potential. According to Riff, this concept and the special scale for its diagnosis (*The Psychological Well-Being Scale*, PWBS) include self-acceptance, positive relations with others, autonomy, environmental mastery, purpose in life, and personal growth. In contrast to mental health, the concept of “psychological well-being” implies a subjective self-perception of the integrity and meaningfulness of an individual’s existence (Ryff et al., 2007).

Research on psychological well-being and ATSMHS shows that for medical students, in addition to the psycho-emotional state, perceiving a need for care is a key stage to start accessing services on the way to well-being (Leão et al., 2011). A cross-cultural study of help-seeking in the United States and Japan shows that, while in the United States, it is associated with high life satisfaction and positive efficacy, in Japan, it is associated with lower well-being scores and low life enjoyment (Lua et al., 2022). Overall, high levels of mental health, low stress, and greater personal flourishing as indicators of psychological well-being are significantly associated with increased resilience and low stigma while help-seeking (Hom et al., 2020). Thartori et al. (2023) revealed that among Albanian immigrant women, telling significant others about their problems increases their sense of security; thus, well-being is positively associated with help-seeking behavior in this subgroup. Özparlak’s meta-analysis showed no significant correlation between mental health literacy and psychological well-being in young people and a low association between literacy and intention to seek help (Özparlak et al., 2023).

Boniwell et al. (2010) conducted a cross-cultural study of the relationship between ZTPI and PWBS subscales in British and Russian student samples. The authors identified five distinct time perspective patterns, four of which were common to both British and Russian students. The balanced time perspective pattern was associated with the highest well-being levels, followed by the hedonistic and future-oriented patterns and risk-taking, while the negative patterns were correlated with low well-being levels. The Balanced time perspective cluster representatives had the highest well-being scores in both samples (Boniwell et al., 2010). However, this study did not examine ATSPPH in relation to time perspectives and well-being patterns.

### 1.3. Mental Health Services and Help-Seeking Attitudes in Russia

While attitudes toward seeking psychological support in Western countries have been actively studied since the mid-20th century, the situation in Russia has remained unique until recent years. Only since the eighties of the 20th century has there been a stage of intensive development of psychotherapy as an independent market and the formation of advisory psychology as a separate branch of psychological science (Marchenkova, 2012). In 2019–2023, the number of specialists in psychological counseling in Russia increased by 18.2% and amounted to 57.7 thousand people (BusinesStat, 2025). From 1 July 2023, by Order of the Russian Minister of Health, medical and psychological support rooms were opened in clinics (Russian Federation Ministry of Health, 2022). Since the 1990s, psychological support offices have been established in schools and universities and, since the 1980s, in some industrial enterprises. With the introduction of the new order, patients facing problems should be able to seek help without waiting for things to worsen. It is now possible to contact a psychologist free of charge as part of compulsory health insurance.

At the beginning of 2025, according to the All-Russian Public Opinion Research Center (VCIOM, 2025), Russia’s leading public opinion research organization, the index of Russians’ need for psychological help was 30 out of 100 (+7 points compared to 2022). Thirteen percent of Russians personally sought professional psychological help (+7 points compared to 2009). In case of problems, every second person turns to their family for advice and help (51%, −11 points compared to 2020), and every fifth person turns to their friends (22%). The VCIOM (2025) study notes that modern Russian youth (especially Zoomers and younger Millennials) grew up in an era of greater accessibility to psychological information, which may explain their higher index of need for psychological help, especially in megacities. The new generation of Russians is more willing to acknowledge their emotional needs and seek help from psychologists; however, in older age groups of Russian society, the culture of seeking professional psychological help is still not widespread (VCIOM, 2025). This also echoes the increased demand for mental health services as one of the top 10 trends in Global Psychology according to the APA (Straight, 2025) and access to qualified mental health professionals as a global challenge according to the 2025 World Economic Forum Annual Meeting (World Economic Forum, 2025).

Thus, we see that today, especially in the post-COVID period, there are both institutional prerequisites and the internal demand of citizens, as well as the development of the market for online and offline services of helping specialists in Russia to provide quality assistance in the field of psychological health. In this regard, the relevance of studying the psychological drivers and barriers for different generations of Russians seeking professional help in the field of mental health increases.

In Russian psychology, the problems of seeking professional psychological help began to be studied much later than in many other countries due to the later development of relevant services. Russian authors examine the issue from the perspective of mass social ideas and social representations point of view, while international scientists more often rely on the concept of attitude. In 2015, Weinstein et al. developed and validated the Russian-language version of the IASMSH (Weinstein et al., 2015), and in 2021, Nesterova and Esipov proposed a Russian-language short version of the ATSPPHS (Nesterova & Esipov, 2021).

Currently, in the Russian-language literature, the differences in seeking psychological help between men and women have been studied in detail (Anikina et al., 2020; Weinstein et al., 2015), and social representations of psychological help among Russian students have also been comprehensively studied (Esipov, 2021; Nesterova & Esipov, 2021). Since 2022, we have been conducting research on ATSPPH factors in samples of Russian university students and have received preliminary results: (1) all PWBS subscales are positively related to the IASMSH total score (Bashkin et al., 2022); (2) students with high scores on the *Autonomy* and *General Self-Efficacy* subscales of the PWBS are inclined to solve psychological problems without seeking professional help (Novikova et al., 2023), and (3) the most significant predictors of the IASMSH total score are PWBS subscales (especially *Personal growth* and *Positive relationships*), time perspective subscales (primarily *Hedonistic present* with a positive impact and *Fatalistic present* with a negative impact), and proactive attitudes by R. Schwarzer (Novikova et al., 2023). In addition, a cross-cultural study on the relationship between PWBS subscales and IASMSH total score among modern Armenian and Russian university students revealed that the differences between the studied samples were manifested not in the absolute severity of the variables, but in the correlations between them (Bashkin et al., 2023).

However, Russian psychologists have not conducted comprehensive and comparative studies examining both the time perspective and psychological well-being in relation to ATSPPH in representatives of different age groups (generations). In the present study, we compared representatives of the Z and Y generations according to W. Strauss and N. Howe Generation Theory (Strauss & Howe, 1991). The adequacy of the Strauss and Howe Generation Theory application on Russian samples has been confirmed by similar Russian studies (Astashova, 2015; Golubinskaya, 2016; Ozhiganova, 2015). The psychological features of representatives of the youngest generation—Generation Z (Zoomers or iGen)—attract special attention from researchers due to the fact that they were born and raised in the “digital era” (Twenge, 2017). One of the central characteristics of this generation, which distinguishes it even from the closest Generation Y, is slower maturation and increasing infantilism (Tikhomandritskaya & Rikel, 2022; Twenge, 2017).

### 1.4. Purpose and Objectives of Present Study

Based on a review of the literature, it can be argued that an important direction in the study of ATSPPH in Russia may be to identify psychological well-being and time perspective as predictors of Y- and Z-Generation differences against the backdrop of the growing growth in psychology services. As we understand, the value of these studies lies in strengthening personal commitment to mental health among young and adult Russians.

The purpose of the present exploratory study was to identify the relationships between ATSMHS indicators, psychological well-being, and time perspective among the Russian Y and Z generations. This study had the following objectives:

(1) Comparison of ATSMHS indicators (IASMHS total score and three subscales), PWBS subscales, and ZTPI subscales between representatives of the Russian Y and Z generations;
(2) Consider and compare the correlations between ATSMHS indicators, psychological well-being, and time perspective subscales among representatives of the Russian Y and Z generations;
(3) Consider the psychological well-being and time perspective subscales as predictors of ATSMHS indicators and compare their impacts in samples of representatives of the Russian Y and Z generations.

Taking into account the review of the literature and results of our previous studies (Bashkin et al., 2022, 2023; Novikova et al., 2023), we assume that there are generational differences in the manifestations of the IASMHS total score and in the associations of this indicator with psychological well-being and time perspective among the Russian Y and Z generations.

## 2. Materials and Methods

### 2.1. Participants

A total of 648 (68% female) Russian respondents aged 17 to 66 years (mean age 25.87) participated in the research. Of the participants, 58% responded that they had experience seeking help from a professional psychologist (only 7% of them regularly received psychological consultations, 31% applied only once, and the rest 2–3 times). The online survey was conducted on the Yandex platform, and data were collected from May to December 2022. The sample was formed using the “snowball” method. Initially, the link to the survey was sent by the authors of the article to their students with a request to participate and, if possible, to invite their friends and relatives. Then a link was provided to potential participants through instant messaging and social networks with the same request to take part in the study and invite their relatives and friends. Participation in this study was voluntary, free, and anonymous; however, the respondents were asked to write their names and email addresses if they agreed to participate in further research. At the end of the survey, by clicking the “send” button, the participants confirmed their consent for the processing of personal data.

Since the initial sample was dominated by representatives of the Y and Z generations, we decided to reduce it using the criterion for inclusion: the absence of missing data and age from 17 to 39 years. A Priori Power Analysis showed that we needed a sample size of approximately 235 participants in each group to reliably (with a probability greater than or equal to 0.9) detect an effect size of *|δ| ≥* 0.3, assuming a two-sided criterion for detection that allowed for a maximum Type I error rate of *α =* 0.05.

In accordance with the purpose of the study, the reduced sample (*N* = 473, 77% female) was divided into two subgroups: Generation Y and Generation Z representatives, according to W. Strauss & N. Howe Generation Theory (Strauss & Howe, 1991).

The Y-Generation sample included 217 (69.9% female) respondents aged 22 to 39 years (mean age 25.80 ± 4.07 years). Most of them are Master’s and PhD students at large Moscow universities.

The Z-Generation sample included 256 (82.8% female) respondents aged 17 to 21 (mean age 19.40 ± 1.13 years). Most of them were undergraduate students at large Moscow universities.

### 2.2. Measures

To achieve the purpose and objectives of this study, we used the following tools.

To diagnose psychological well-being, The Psychological Well-Being Scale (PWBS) by Ryff (1989) in Russian localization by Shevelenkova and Fesenko (https://smarteka.com/uploads/files/2020/05/26/a90887d7-b78d-4d81-95cb-de5641fc33cb43b07ad8-e9a8-49ac-bc58-6ad9c45de2cf.pdf assessed 25 February 2005) was used. This Scale consists of 84 items and includes 6 subscales: *self-acceptance, positive relations with others, autonomy, environmental mastery, purpose in life,* and *personal growth*. The items were rated on a 6-point Likert scale (1 = strongly disagree to 6 = strongly agree). Scores are calculated for each subscale as well as for the scale as a whole (indicator of general psychological well-being). In our study, all the subscales of the PWBS had good Cronbach’s α (0.79–0.83) and McDonald’s ω (0.79–0.83) coefficients.

To determine the time perspective, we used the Zimbardo Time Perspective Inventory (ZTPI) (Zimbardo & Boyd, 1999), which was adopted in Russia by Sircova et al. (2008). It aims to study the relationship of the individual with time (past, present, and future). The ZTPI consists of 56 items and includes five subscales: negative and positive past, hedonistic and fatalistic present, and future. The items were rated on a 5-point Likert scale (1 = absolutely wrong to 5 = absolutely right). In our study, all the subscales of the ZTPI had acceptable Cronbach’s α (0.76–0.79) and McDonald’s ω (0.77–0.80) coefficients.

To reveal ATSMHS, the Inventory of Attitudes toward Seeking Mental Health Services (IASMHS), adapted in Russia by Weinstein et al. (2015), was used. The questionnaire consists of 24 items and includes three subscales: (1) indifference to stigma, (2) propensity to seek help, and (3) psychological openness. IASMHS total score allows for the assessment of the respondent’s attitude toward seeking psychological help. The items were rated on a 5-point Likert scale (1 = disagree to 5 = agree). In our study, the IASMHS indicators have acceptable and high Cronbach’s and McDonald’s coefficients: α = 0.91 and ω = 0.91 for total score; α = 0.86 and ω = 0.87 for *indifference to stigma* subscale; α = 0.83 and ω = 0.83 for *propensity to seek help* subscale; α = 0.78 and ω = 0.78 for *psychological openness* subscale.

The survey also included several questions to collect *sociodemographic data*, such as gender, age, occupation (study/work), and experience seeking psychological help.

### 2.3. Statistical Analysis

The R software environment for statistical computing and graphics, version 4.1.1 (R Core Team, 2025; Revelle, 2025; The Jamovi Project, 2025), was used for statistical processing. The descriptive statistics, Cronbach’s *α* and McDonald’s *ω* coefficients, Spearman’s rank correlation analysis, Mann-Whitney’ U-Test, Fisher *F*-test, and regression analysis (“backward” stepwise search for “a best predictor model”) were used for statistical analysis. The independent variables were the six PWBS subscales and five ZTPI scales, and the dependent variables were the ATSMHS indicators (IASMHS total score and three subscales). A Priori Power Analysis and Power by Effect Size calculation were performed using jpower 0.1.3 module to compute power for various designs within jamovi ((https://github.com/richarddmorey/jpower, accessed on 25 February 2025). Cohen’s d for Mann-Whitney’*U*-Test was calculated from z-value according to Lenhard and Lenhard (2022).

## 3. Results

### 3.1. Descriptive Statistics and Analysis of Differences

The results of the descriptive statistics and analysis of differences are presented in Table 1.

Table 1 shows higher scores for all PWBS subscales and the total psychological well-being score in the Y-Generation sample than in the Z-Generation participants. However, the difference was statistically significant only for the *Autonomy* scale (*p* = 0.029) and for the *Purpose in life* scale at the level of statistical tendency (*p* = 0.066). No significant differences were found in the severity of the ZTPI and IASMHS subscales and total scores between the Y-Generation- and Z-Generation samples. However, it should be noted that *Psychological openness* is slightly higher in the Z-Generation at the tendency level (*p* = 0.057).

### 3.2. Correlation Analysis

Spearman’s correlations between PWBS subscales and total score, ZTPI subscales, and IASMHS subscales and total score are presented in Table 2, Table 3 and Table 4.

Table 2 shows that the IASMHS subscales and total score have significant positive correlations with all PWBS subscales, as well as *Hedonistic present* (except for a non-significant correlation with *Psychological Openness*), *Future,* and *Positive past* in the general sample. *Negative past* and *Fatalistic present* were also significantly negatively correlated with the subscales and total score of the IASMHS.

The IASMHS total score and *Indifference to Stigma* and *Help-seeking Propensity* subscales had positive significant correlations with all PWBS subscales, *Hedonistic present, Future,* and *Positive past* in Y-Generation as well and Z-Generation samples. *Negative past* and *Fatalistic present* were significantly negatively correlated with the total score of the IASMHS in these samples (Table 3 and Table 4). The exception in the Z-Generation is the absence of similar significant correlations between *Psychological Openness* with *Hedonistic present* and *Future* (ZTPI subscales). In contrast, the *Psychological Openness* subscale did not have significant positive correlations with *Autonomy* and *Environmental mastery* (PWBS subscales) and *Hedonistic present* and *Future* (ZTPI subscales) in Y-Generation (Table 4).

### 3.3. Regression Analysis

The results of the linear regression analysis are presented in Table 5, Table 6, Table 7, Table 8, Table 9, Table 10, Table 11, Table 12, Table 13, Table 14, Table 15 and Table 16. All full and best predictor models with the IASMHS subscales and total score as the dependent variable and the PWBS and ZTPI subscales as predictors were statistically valid according to the Fisher *F*-test. Therefore, we can state that these predictors have a significant impact on the ATSMHS indicator. It should be noted that, since this study has a cross-sectional design, we use the term “predictor” only as a statistical concept within the framework of regression analysis.

The best predictor model for the IASMHS total score explained 37.3% of the variance in the general sample: *Personal growth* and *Positive relations* (PWBS subscales) as well as *Positive past* and *Hedonistic present* (ZTPI subscales) had a significant positive impact, and *Fatalistic present* (ZTPI subscale) had a significant negative impact (Table 5).

The best predictor model for the IASMHS total score explains 38.5% of the variance in the Z-Generation: *Personal growth* (PWBS subscale) has a significant positive impact, and *Fatalistic present* (ZTPI subscale) has a significant negative impact (Table 6).

The best predictor model for the IASMHS total score explained 40.3% of the variance in the Y-Generation: *Personal growth* and *Positive relations* (PWBS subscales) as well as *Hedonistic present* (ZTPI subscale) had a significant positive impact, and *Fatalistic present* (ZTPI subscale) had a significant negative impact (Table 7).

The best predictor model for the IASMHS *Indifference to Stigma* subscale explained 37.4% of the variance in the general sample: *Personal growth* and *Positive relations* (PWBS subscales) as well as *Positive past* and *Hedonistic present* (ZTPI subscales) had a significant positive impact, and *Environmental mastery* (PWBS subscale) and *Fatalistic present* (ZTPI subscale) had a significant negative impact (Table 8).

The best predictor model for the IASMHS *Indifference to Stigma* subscale explained 39.9% of the variance in the Z-Generation: *Personal growth* and *Positive relations* (PWBS subscales) as well as Hedonistic present (ZTPI subscale) had a significant positive impact, and *Fatalistic present* (ZTPI subscale) had a significant negative impact (Table 9).

The best predictor model for the IASMHS *Indifference to Stigma* subscale explains 37.4% of the variance in the Y-Generation: *Personal growth* and *Positive relations* (PWBS subscales), as well as *Hedonistic present* (ZTPI subscale), have a significant positive impact, and *Negative past* and *Fatalistic present* (ZTPI subscales) have a significant negative impact (Table 10).

The best predictor model for the IASMHS *Help-seeking Propensity* subscale explained 32.5% of the variance in the general sample: *Personal growth* and *Environmental mastery* (PWBS subscales), as well as *Future* and *Hedonistic present* (ZTPI subscales) had a significant positive impact (Table 11).

The best predictor model for the IASMHS *Help-seeking Propensity* subscale explained 31.0% of the variance in the Z-Generation: *Personal growth* (PWBS subscale) and *Future* (ZTPI subscale) had a significant positive impact (Table 12).

The best predictor model for the IASMHS *Help-seeking Propensity* subscale explained 35.2% of the variance in the Y-Generation: *Personal growth* and *Environmental mastery* (PWBS subscales) as well as *Hedonistic present* (ZTPI subscale) had a significant positive impact, and *Autonomy* (PWBS subscale) had a significant negative impact (Table 13).

The best predictor model for IASMHS *Psychological openness* explained only 20.0% of the variance in the general sample: *Personal growth* and *Positive relations* (PWBS subscales) had a significant positive impact, and *Fatalistic present* (ZTPI subscale) had a significant negative impact (Table 14).

The best predictor model for IASMHS *Psychological openness* explains 22.0% of the variance in the Z-Generation: *Personal growth* (PWBS subscale) and *Future* (ZTPI subscale) have a significant positive impact, and Environmental mastery (PWBS subscale) and *Fatalistic present* (ZTPI subscale) have a significant negative impact (Table 15).

The best predictor model for IASMHS *Psychological openness* explained 24,3% of the variance in the Y-Generation: *Personal growth* and *Positive relations* (PWBS subscales) as well as *Hedonistic present* (ZTPI subscale) had a significant positive impact, and *Environmental mastery* (PWBS subscale) and *Fatalistic present* (ZTPI subscale) had a significant negative impact (Table 16).

## 4. Discussion

The present exploratory cross-sectional study aimed to identify and compare the relationship of the ATSMHS with psychological well-being and time perspective indicators between Russian Y- and Z-Generation representatives. Based on the literature review and our previous ATSMHS studies, we assumed that indicators of psychological well-being and time perspective may be correlates and predictors of ATSMHS, but the severity and associations of the variables studied may differ between Generation Y and Generation Z. However, based on the results of the study, our assumptions were only partially confirmed.

To answer the first research question, we compared ATSMHS indicators (IASMHS total score and three subscales: *Indifference to Stigma, Help-seeking Propensity,* and *Psychological openness*), PWBS, and ZTPI subscales between representatives of the Russian Y and Z generations. Contrary to our expectations, we found only one statistically significant difference between all variables studied: the *Autonomy* subscale of the PWBS had higher scores in the Y-Generation representatives. On the one hand, both generations studied are characterized by life during a period of reassessment of values in society, as well as increasingly individualized digitalization. On the other hand, the ability to think and act independently of social pressure as strictly opposed to their parental involvement in large social groups is more common among the Y-Generation, while Z-Generation representatives who grew up without attacks on their own opinions are more spontaneous and natural (Trenina et al., 2022). When seeking professional psychological help, Z-Generation does not confront but rather trustingly relies on the opinion of their parents (Nesterova & Esipov, 2021; Twenge, 2017). This also correlates with the VCIOM (2025) assessment that Millennials were brought up more strictly with self-reliance and some prohibitions on asking for help outside the family.

At the same time, the lack in differences of the time perspective scales between the Y and Z Generations is entirely consistent with A. Gonzales’ and P.G. Zimbardo’s initial position that time perspective is a stable framework for the existence of a person’s mental life (Gonzales & Zimbardo, 1985). Previously contradictory data were obtained regarding generational differences in the severity of ATSMHS (Picco et al., 2016): on the one hand, younger respondents have a greater openness to seeking psychological help (Rickwood et al., 2005), while on the other hand, older respondents were more interested in seeking help due to the presence of more mental health problems (Ropret et al., 2023). The greater psychological openness for psychological help in the more youthful generation was confirmed in our study at a level close to significance.

The answer to the second research question showed that the correlations of ATSMHS indicators with both psychological well-being and time perspective were generally consistent across the samples studied (general, Y, and Z samples). However, small differences in the correlation significance of *Psychological Openness* with PWBS and ZTPI subscales can be noted: fewer significant correlations were obtained for the Y-Generation sample. Previous research has shown that the association between ATSMHS and psychological well-being may vary across cross-cultural studies. For example, Lua et al. (2022) showed that help-seeking tendencies were associated with greater life satisfaction and higher positive affect among Americans, but these tendencies were associated with lower life satisfaction and lower positive affect among the Japanese. Bashkin et al. (2023), in a study of representatives of Z-Generation, found that ATSMHS were positively related to all PWBS subscales in Russian university students, but ATSMHS were weakly related to these subscales (with the exception of *Personal growth*) in Armenian university students. Özparlak’s meta-analysis showed no correlation between mental health literacy and mental well-being in young people (Özparlak et al., 2023). Therefore, our data confirm strong positive correlations between ATSMHS indicators and all PWBS indicators not only in the Z-Generation representatives but also in the older sample of Y-Generation representatives in Russia. In our opinion, these findings highlight the issue of maintaining, preserving, and preventing mental health, as respondents with potentially more severe problems with psychological well-being are generally less likely to seek professional help in the field of mental health, which may make it more difficult to resolve existing problems. The time perspective as a correlate and predictor of ATSMHS has been less studied, but the few studies that do exist do not seem to support the theoretically expected relationships between time perspective and ATSMHS (Kiss et al., 2020). However, our data confirm the theoretical expectations of Kiss et al. (2020) that the future time perspective may be a facilitating factor and the fatalistic present-time perspective may be an inhibitory factor for seeking professional psychological help. Overall, our results are consistent with previous research findings that positive past experiences (Rickwood et al., 2005), long-term (future) orientation (Koon et al., 2023), and partly positive present (for mostly older individuals) (Erickson et al., 2017) are significantly and positively related to mental health help-seeking.

Finally, addressing the third research question, which considered and compared the PWBS and ZTPI indicators as predictors of ATSMHS indicators, allowed us to identify the similarities and differences between the Y and Z generations. Firstly, the similarity lies in the fact that in both generations, the combination of some PWBS and ZTPI scales explains 37.3–40.3% of the IASMHS total score variance, 37.4–39.9% of the *Indifference to Stigma* variance, 31.0–35.2% of the *Help-seeking Propensity,* and 20.0–24.3% of the *Psychological openness* variance. It is important to note that the highest rates of explained variance were obtained for both the IASMHS total score and the *Indifference to Stigma* subscale, which is considered one of the most important factors determining the seeking of psychological help (Z.-X. Chen & Chandrasekara, 2016; Kiss et al., 2020; Nwankwo et al., 2019; Rayan & Jaradat, 2016; Shahwan et al., 2020; Swisher et al., 2024; Yasmin et al., 2024; Zhou et al., 2019). We believe that these determination coefficients are high given the many factors that influence ATSMHS, and these coefficients also confirm our initial assumption that psychological well-being and time perspective have a significant impact on ATSMHS indicators.

Secondly, the similarity between generations is manifested in the fact that the *Personal growth* (PWBS subscale) is a positive predictor, and the *Fatalistic present* (ZTPI subscale) is a negative predictor of the IASMHS total score. These predictors (in combination with others) were significant for both *Indifference to Stigma* and *Psychological openness* in all samples studied. Only *Personal growth* was a positive predictor of *Help-seeking Propensity* in both generations. Thirdly, similarities include the almost complete coincidence of significant predictors for *Indifference to Stigma* in both generations (*Personal growth, Positive relations,* and *Hedonistic present* are positive predictors, and *Fatalistic present* is a negative predictor).

These results allow us to clarify the conclusions of the correlation analysis: more positive attitudes toward seeking psychological help are facilitated by *Personal growth* among the components of psychological well-being and inhibited by the *Fatalistic Present* as the dominant time perspective. Thus, individuals who are focused on gaining new experiences and, self-realization and self-development and who do not consider their current life to be predetermined and impossible to change are more likely to seek and receive professional help regarding mental health. These findings are consistent with the general tenets of Ryff’s concept of psychological well-being (Ryff, 1989; Ryff et al., 2007) and Zimbardo’s time perspective framework (Gonzales & Zimbardo, 1985; Zimbardo & Boyd, 1999). More specifically, our results support the findings on the important role of *Personal growth* in Russian-speaking samples (Bashkin et al., 2022, 2023; Novikova et al., 2023), as well as conceptual assumptions about the inhibitory influence of the *Fatalistic present* on ATSMHS (Kiss et al., 2020).

Differences between generations lie in the additional significant predictors of ATSMHS indicators. In most cases, including the IASMHS total score, positive predictors for Y-Generation were *Positive relations* and *Hedonistic present* and *Future* for Z-Generation. The data obtained are related to the need to maintain positive emotions in a predictable and reliable social environment through psychological assistance in Generation Y, which has been familiar with periods of social instability since childhood. For Generation Z, the predictors of seeking psychological help are the unpredictability of the future and the lack of successful personal experience by living through unsafe instability periods (Trenina et al., 2022). As studies show, future-oriented people are already in a good physical and mental shape (Kiss et al., 2020), but for Generation Z, this trend is becoming an independent indicator for prolonging this state into the future, investing in their own human capital (World Economic Forum, 2025), when it is not the content only that is remembered, but rather the place (Nesterova & Esipov, 2021) and affiliated people associated with the information found (Sapa, 2014).

Promoting personal growth and reducing fatalistic attitudes could encourage mental health service utilization in Russian society by working to improve psychological literacy and reduce the fear of social isolation and the risks of stigmatization (Z.-X. Chen & Chandrasekara, 2016). Individual preferences in programs used must be strongly considered in the unique needs of both generations (Sheikh et al., 2024). Psychoeducation can reduce stigmatizing attitudes through scenario-based teaching relevant to generational time perspective characteristics (Waqas et al., 2020). For Y-Generation representatives, with an inward focus, autonomy, and psychoeducational programs aimed at maintaining a positive state and reducing possible social stigma will be more effective. The lack of ego control in future orientation with lower autonomy in representatives of Generation Z makes it necessary to form greater self-reliance and reduce self-stigmatization as work targets.

When assessing the findings of this study, its *limitations* must also be taken into account. The limitations of our study primarily lie in the gender, age, and educational composition of the samples: (1) both samples are dominated by females, (2) both samples are highly educated, and (3) the mean age gap between the samples is not very large. Other limitations are that we used only self-reported data, convenient snowball sampling, and a cross-sectional study design. It should also be noted that the study was conducted during a difficult period associated with the end of the pandemic and the growth of international tension and conflict.

## 5. Conclusions

Research on attitudes toward seeking psychological help or professional help in the field of mental health has occupied an important place among studies on ways to maintain and strengthen the mental health of the population in different countries for more than half a century. The findings of these studies allow us to identify sociodemographic, social, psychological, and other factors that contribute to and hinder seeking help for mental health and, accordingly, to outline ways to overcome existing barriers. In Russia, such studies began relatively recently, at the beginning of the 21st century, and have become especially popular during the COVID-19 pandemic and in the post-pandemic period.

In this study, psychological well-being and time perspective were considered psychological correlates and predictors of ATSMHS among representatives of the Y and Z generations. Analysis of the research results allows us to draw the following conclusions in accordance with the research objectives.

- No significant differences were found between representatives of the Y and Z generations in ATSMHS, time perspective, and psychological well-being (with the exception of one subscale).
- Stable positive correlations were confirmed between all subscales of psychological well-being and ATSMHS indicators among representatives of the Y and Z generations. At the same time, it was established that the *Negative past* and *Fatalistic present* were correlated negatively with ATSMHS indicators, and the remaining scales of time perspective correlated positively among representatives of both generations.
- It was revealed that *Personal growth* is a positive predictor and *Fatalistic present* is a negative predictor for most ATSMHS indicators in samples of both generations. The differences between generations are that additional significant positive predictors for ATSMHS indicators for Y-Generation are *Positive relations* and *Hedonistic present*, and for Z-Generation—only *Future* time perspective;
- The findings of our study are important for developing programs to maintain, preserve and prevent mental health across generations. Psychological education and support programs aimed at promoting personal growth, developing one’s potential, increasing interest in life, overcoming boredom, and reducing belief in a fatalistic and predetermined present will contribute to strengthening the tendency to seek help in the field of mental health among representatives of both the Y and Z generations. Additionally, it is important to help Y-Generation representatives focus on building trusting relationships with others and being able to enjoy the present, while Z-Generation needs to be encouraged to develop ego control in future orientation.

Thus, we can state that, in general, contrary to our assumptions, in the relationships between the ATSMHS and psychological well-being and time perspective among representatives of the Y and Z generations in our study, similarities prevail. This may be explained by the fact that the respondents belong to the same culture, but verification of this assumption requires additional cross-cultural studies.

Despite some limitations, the present study is one of the first in Russian psychology to comprehensively examine psychological well-being and time perspective as correlates and predictors of ATSMHS across generations. The findings of this study may serve as a starting point for discussions and further developments in this field.

The prospects for ***further research*** are associated with (1) balancing the sample by the female-to-male ratio; (2) expanding the sample by representatives of other generations; (3) using additional methods for ATSMHS indicator measurement (for example, expert assessment); (4) exploring other correlates and possible predictors of ATSMHS (e.g., coping strategies, personality traits, proactive attitudes); (5) conducting cross-cultural studies using analogous tools for diagnosing the variables being studied.

## Figures and Tables

**Table 1:** Means (M), standard deviations (SD), and Mann−Whitney’ *U*-Test between study variables in Z-Generation and Y-Generation participants.

Variables	General Sample		Z-Gen		Y-Gen		Mann-Whitney’	*p*-Level	Cohen’s
	(*N* = 473)		(*N* = 256)		(*N* = 217)		*U*-Test		d
	M	SD	M	SD	M	SD			
Psychological Well-Being (PWBS subscales)									
Positive relations	58.72	10.07	57	10.31	58	9.78	25,957	0.219	0.113
Autonomy	57.58	10.52	55.5	10.6	59	10.38	24,538	0.029 *	0.202
Environmental	55.99	9.25	54.5	9.47	56	8.96	25,746	0.17	0.126
mastery									
Personal growth	63.24	10.03	64	10.08	65	9.96	26,025	0.237	0.109
Purpose in life	60.55	10.47	59	10.58	62	10.29	25,056	0.066	0.165
Self-acceptance	57.58	11.02	57	10.96	58	11.12	27,769	0.996	0
Psychological	353.66	50.64	351	51.92	361	48.98	25,681	0.157	0.13
Well-Being									
(total score)									
Time Perspective (ZTPI subscales)									
Negative past	29	7.84	29	7.69	29	8.04	27,239	0.717	0.033
Hedonistic present	51.4	7.24	51	7.18	51	8.04	26,231	0.296	0.096
Future	45.99	7.37	45	7.54	46	7.15	25,461	0.118	0.144
Positive past	31.63	5.69	31	5.67	32	5.72	26,279	0.311	0.093
Fatalistic present	25.13	5.99	25	5.94	25	6.08	27,468	0.835	0.019
Attitudes toward Seeking Mental Health Services									
Indifference to	32	6.93	31.5	6.81	32	7.07	27,201	0.697	0.036
Stigma									
Help-seeking	30	6.21	30	6.23	31	6.19	27,418	0.809	0.022
Propensity									
Psychological	28	6.25	29	5.92	27	6.57	24,961	*0.057*	0.175
openness									
IASMHS	91	16.53	91	16.17	89	16.96	26,655	0.449	0.07
total score									

* *p* < 0.05; *p* ≤ 0.10—in italic.

**Table 2:** Spearman’s correlations between studied variables in the general sample of two generations’ participants (*N* = 473).

Variables	1	2	3	4	5	6	7	8	9	10	11	12	13	14	15	16
1. PR	—															
2. A	0.44 ***	—														
3. EM	0.602 ***	0.533 ***	—													
4. PG	0.612 ***	0.556 ***	0.587 ***	—												
5. PiL	0.636 ***	0.526 ***	0.747 ***	0.787 ***	—											
6. S-A	0.600 ***	0.586 ***	0.729 ***	0.64 ***	0.731 ***	—										
7. W-B	0.776 ***	0.731 ***	0.837 ***	0.836 ***	0.887 ***	0.866 ***	—									
8. NP	−0.469 ***	−0.467 ***	−0.578 ***	−0.382 ***	−0.473 ***	−0.702 ***	−0.621 ***	—								
9. HP	0.239 ***	0.202 ***	0.114	0.301 ***	0.184 ***	0.193 ***	0.249 ***	0.031	—							
10. F	0.377 ***	0.209 ***	0.486 ***	0.47 ***	0.58 ***	0.314 ***	0.476 ***	−0.118 *	0.098	—						
11. PP	0.498 ***	0.256 ***	0.453 ***	0.358 ***	0.457 ***	0.464 ***	0.498 ***	−0.381 ***	0.270 ***	0.315 ***	—					
12. FP	−0.373 ***	−0.387 ***	−0.465 ***	−0.486 ***	−0.518 ***	−0.441 ***	−0.53 ***	0.46 ***	0.183 **	−0.318 ***	−0.143 *	—				
13. IS	0.496 ***	0.418 ***	0.405 ***	0.537 ***	0.486 ***	0.471 ***	0.563 ***	−0.369 ***	0.198 ***	0.26 ***	0.346 ***	−0.443 ***	—			
14. H-SP	0.430 ***	0.286 ***	0.447 ***	0.526 ***	0.512 ***	0.439 ***	0.529 ***	−0.259 ***	0.278 ***	0.397 ***	0.328 ***	−0.310 ***	0.570 ***	—		
15. PO	0.297 ***	0.202 ***	0.196 ***	0.397 ***	0.339 ***	0.250 ***	0.333 ***	−0.185 ***	0.114	0.247 ***	0.182 **	−0.349 ***	0.640 ***	0.614 ***	—	
16. IAS	0.480 ***	0.351 ***	0.405 ***	0.566 ***	0.52 ***	0.45 ***	0.553 ***	−0.317 ***	0.221 ***	0.348 ***	0.329 ***	−0.428 ***	0.863 ***	0.833 ***	0.872 ***	—

* *p* ≤ 0.05; ** *p* ≤ 0.01; *** *p* ≤ 0.001, corrected with Holm correction method. PR—Positive relations; A—Autonomy; EM—Environmental mastery; PG—Personal growth; PiL—Purpose in life; S-A—Self-acceptance; W-B—Psychological Well-Being (total score); NP—Negative past; HP—Hedonistic present; F—Future; PP—Positive past; FP—Fatalistic present; IS—Indifference to Stigma; H-SP—Help-seeking Propensity; PO—Psychological openness; IAS—IASMHS total score.

**Table 3:** Spearman’s correlations between studied variables in the Z-Generation sample (*N* = 256).

Variables	1	2	3	4	5	6	7	8	9	10	11	12	13	14	15	16
1. PR	—															
2. A	0.490 ***	—														
3. EM	0.594 ***	0.529 ***	—													
4. PG	0.627 ***	0.583 ***	0.562 ***	—												
5. PiL	0.641 ***	0.538 ***	0.726 ***	0.788 ***	—											
6. S-A	0.626 ***	0.622 ***	0.714 ***	0.642 ***	0.719 ***	—										
7. W-B	0.795 ***	0.750 ***	0.815 ***	0.837 ***	0.883 ***	0.868 ***	—									
8. NP	−0.433 ***	−0.418 ***	−0.536 ***	−0.371 ***	−0.459 ***	−0.698 ***	−0.581 ***	—								
9. HP	0.324 ***	0.273 ***	0.21 **	0.378 ***	0.278 ***	0.255 ***	0.340 ***	0.042	—							
10. F	0.363 ***	0.228 ***	0.480 ***	0.469 ***	0.583 ***	0.333 ***	0.483 ***	−0.124	0.186 *	—						
11. PP	0.484 ***	0.197 ***	0.466 ***	0.305 ***	0.438 ***	0.454 ***	0.462 ***	−0.307 ***	0.248 ***	0.317 ***	—					
12. FP	−0.386 ***	−0.422 ***	−0.501 ***	−0.539 ***	−0.558 ***	−0.481 ***	−0.575 ***	0.486 ***	0.074	−0.348 ***	−0.166	—				
13. IS	0.516 ***	0.441 ***	0.421 ***	0.569 ***	0.518 ***	0.496 ***	0.600 ***	−0.314 ***	0.192 *	0.285 ***	0.342 ***	−0.450 ***	—			
14. H-SP	0.406 ***	0.306 ***	0.439 ***	0.519 ***	0.515 ***	0.412 ***	0.525 ***	−0.212 ***	0.279 ***	0.449 ***	0.283 ***	−0.346 ***	0.589 ***	—		
15. PO	0.251 ***	0.233 ***	0.237 **	0.407 ***	0.383 ***	0.259 ***	0.364 ***	−0.160	0.062	0.347 ***	0.145	−0.378 ***	0.648 ***	0.598 ***	—	
16. IAS	0.463 ***	0.384 ***	0.433	0.584 ***	0.558 ***	0.458 ***	0.584 ***	−0.273 ***	0.198 **	0.413 ***	0.300 ***	−0.457 ***	0.873 ***	0.835 ***	0.862 ***	—

* *p* ≤ 0.05; ** *p* ≤ 0.01; *** *p* ≤ 0.001, corrected with Holm correction method. PR—Positive relations; A—Autonomy; EM—Environmental mastery; PG—Personal growth; PiL—Purpose in life; S-A—Self-acceptance; W-B—Psychological Well-Being (total score); NP—Negative past; HP—Hedonistic present; F—Future; PP—Positive past; FP—Fatalistic present; IS—Indifference to Stigma; H-SP—Help-seeking Propensity; PO—Psychological openness; IAS—IASMHS total score.

**Table 4:** Spearman’s correlations between studied variables in the Y-Generation sample (*N* = 217).

Variables	1	2	3	4	5	6	7	8	9	10	11	12	13	14	15	16
1. PR	—															
2. A	0.372 ***	—														
3. EM	0.612 ***	0.522 ***	—													
4. PG	0.594 ***	0.508 ***	0.605 ***	—												
5. PiL	0.624 ***	0.486 ***	0.765 ***	0.784 ***	—											
6. S-A	0.57 ***	0.542 ***	0.743 ***	0.636 ***	0.748 ***	—										
7. W-B	0.755 ***	0.695 ***	0.856 ***	0.832 ***	0.889 ***	0.870 ***	—									
8. NP	−0.513 ***	−0.528 ***	−0.623 ***	−0.39 ***	−0.492 ***	−0.700 ***	−0.668 ***	—								
9. HP	0.148	0.114	0.015	0.215 *	0.079	0.13	0.148	0.014	—							
10. F	0.382 ***	0.175	0.479 ***	0.465 ***	0.557 ***	0.28 ***	0.45 ***	−0.112	−0.007	—						
11. PP	0.503 ***	0.317 ***	0.419 ***	0.405 ***	0.466 ***	0.47 ***	0.525 ***	−0.459 ***	0.295	0.311 ***	—					
12. FP	−0.355 ***	−0.348 ***	−0.430 ***	−0.419 ***	−0.477 ***	−0.4 ***	−0.479 ***	0.427 ***	0.3 ***	−0.275 ***	−0.109	—				
13. IS	0.475 ***	0.392 ***	0.396 ***	0.501 ***	0.459 ***	0.44 ***	0.525 ***	−0.429 ***	0.202 **	0.226 ***	0.353 ***	−0.432 ***	—			
14. H-SP	0.457 ***	0.252 ***	0.460 ***	0.531 ***	0.514 ***	0.47 ***	0.539 ***	−0.312 ***	0.278 ***	0.330 ***	0.376 ***	−0.271 **	0.554 ***	—		
15. PO	0.360 ***	0.190	0.172	0.394 ***	0.312 ***	0.240 **	0.321 ***	−0.217 *	0.164	0.143	0.229 **	−0.319 ***	0.638 ***	0.642 ***	—	
16. IAS	0.501 ***	0.317 ***	0.389 ***	0.542 ***	0.490 ***	0.440 ***	0.527 ***	−0.365 ***	0.240 **	0.273 ***	0.361 ***	−0.397 ***	0.852 ***	0.833 ***	0.884 ***	—

* *p* ≤ 0.05; ** *p* ≤ 0.01; *** *p* ≤ 0.001, corrected with Holm correction method. PR—Positive relations; A—Autonomy; EM—Environmental mastery; PG—Personal growth; PiL—Purpose in life; S-A—Self-acceptance; W-B—Psychological Well-Being (total score); NP—Negative past; HP—Hedonistic present; F—Future; PP—Positive past; FP—Fatalistic present; IS—Indifference to Stigma; H-SP—Help-seeking Propensity; PO—Psychological openness; IAS—IASMHS total score.

**Table 5:** Full and best predictor regression models for the IASMHS total score in the general sample of two generations of participants (*N* = 473).

“Predictors”	Summary of Model				Coefficients		
	R^2^_adj_	*F*	*p*-Value	Estimate	Std. Error	*t*-Value	*p*-Value
**Full model**	0.377	26.9	<0.001				
Constant				46.026	9.268	4.966	<0.001
Positive relations				0.229	0.086	2.650	0.008
Autonomy				−0.059	0.078	−0.754	0.451
Environmental mastery				−0.192	0.117	−1.636	0.102
Personal growth				0.524	0.109	4.816	<0.001
Purpose in life				0.093	0.124	0.748	0.455
Self-acceptance				−0.037	0.109	−0.341	0.734
Negative past				−0.137	0.127	−1.084	0.279
Hedonistic present				0.261	0.101	2.587	0.010
Future				0.122	0.110	1.113	0.266
Positive past				0.244	0.131	1.873	0.062
Fatalistic present				−0.615	0.139	−4.420	<0.001
**Best predictor model**	0.373	71.2	<0.001				
Constant				41.069	6.589	6.23	<0.001
Positive relations				0.271	0.077	3.52	<0.001
Personal growth				0.542	0.087	6.24	<0.001
Hedonistic present				0.259	0.098	2.64	0.009
Fatalistic present				−0.610	0.128	−4.75	<0.001

**Table 6:** Full and best predictor regression models for the IASMHS total score in the Z-Generation sample (*N* = 256).

“Predictors”	Summary of Model				Coefficients		
	R^2^_adj_	*F*	*p*-Value	Estimate	Std. Error	*t*-Value	*p*-Value
**Full model**	0.378	15.1	<0.001				
Constant				38.183	12.079	3.161	0.002
Positive relations				0.158	0.118	1.344	0.180
Autonomy				0.014	0.111	0.122	0.903
Environmental mastery				−0.212	0.149	−1.421	0.157
Personal growth				0.620	0.147	4.214	<0.001
Purpose in life				0.13	0.159	0.814	0.416
Self-acceptance				−0.030	0.156	−0.194	0.847
Negative past				−0.016	0.169	−0.094	0.925
Hedonistic present				−0.039	0.137	−0.285	0.776
Future				0.259	0.144	1.800	0.073
Positive past				0.280	0.176	1.591	0.113
Fatalistic present				−0.380	0.193	−1.967	0.050
**Best predictor model**	0.385	40.9	<0.001				
Constant				35.761	9.794	3.65	<0.001
Personal growth				0.705	0.100	7.01	<0.001
Future				0.237	0.122	1.95	0.053
Positive past				0.280	0.151	1.86	0.065
Fatalistic present				−0.384	0.159	−2.41	0.017

**Table 7:** Full and best predictor regression models for the IASMHS total score in the Y-Generation sample (*N* = 217).

“Predictors”	Summary of Model				Coefficients		
	R^2^_adj_	*F*	*p*-Value	Estimate	Std. Error	*t*-Value	*p*-Value
**Full model**	0.392	13.6	<0.001				
Constant				45.916	14.969	3.067	0.002
Positive relations				0.312	0.130	2.399	0.017
Autonomy				−0.075	0.115	−0.652	0.515
Environmental mastery				−0.129	0.192	−0.672	0.502
Personal growth				0.436	0.162	2.691	0.008
Purpose in life				0.096	0.197	0.485	0.628
Self-acceptance				−0.075	0.158	−0.474	0.636
Negative past				−0.213	0.200	−1.066	0.288
Hedonistic present				0.55	0.153	3.592	<0.001
Future				−0.005	0.171	−0.027	0.979
Positive past				0.177	0.204	0.866	0.388
Fatalistic present				−0.838	0.204	−4.108	<0.001
**Best predictor model**	0.403	37.4	<0.001				
Constant				32.477	9.535	3.41	<0.001
Positive relations				0.374	0.113	3.32	0.001
Personal growth				0.412	0.121	3.42	<0.001
Hedonistic present				0.567	0.145	3.91	<0.001
Fatalistic present				−0.856	0.188	−4.56	<0.001

**Table 8:** Full and best predictor regression models for *Indifference to Stigma* in the general sample of two generations’ participants (N = 473).

Predictors	Summary of Model				Coefficients		
	R^2^_adj_	*F*	*p*-Value	Estimate	Std. Error	*t*-Value	*p*-Value
**Full model**	0.372	26.4	<0.001				
Constant				17.015	3.898	4.365	<0.001
Positive relations				0.128	0.036	3.522	<0.001
Autonomy				0.042	0.033	1.276	0.202
Environmental mastery				−0.085	0.049	−1.717	0.087
Personal growth				0.180	0.046	3.937	<0.001
Purpose in life				0.012	0.052	0.229	0.819
Self-acceptance				−0.013	0.046	−0.288	0.773
Negative past				−0.059	0.053	−1.108	0.268
Hedonistic present				0.073	0.042	1.720	0.086
Future				−0.033	0.046	−0.705	0.481
Positive past				0.130	0.055	2.377	0.018
Fatalistic present				−0.300	0.059	−5.123	<0.001
**Best predictor model**	0.374	41.3	<0.001				
Constant				18.026	3.539	5.093	<0.001
Positive relations				0.128	0.036	3.544	<0.001
Environmental mastery				−0.087	0.041	−2.109	0.036
Personal growth				0.189	0.038	4.993	<0.001
Negative past				−0.079	0.043	−1.845	0.066
Hedonistic present				0.083	0.042	1.998	0.046
Positive past				0.117	0.054	2.177	0.030
Fatalistic present				−0.302	0.057	−5.302	<0.001

**Table 9:** Full and best predictor regression models for *Indifference to Stigma* in Z-Generation sample (N = 256).

“Predictors”	Summary of Model				Coefficients		
	R^2^_adj_	*F*	*p*-Value	Estimate	Std. Error	*t*-Value	*p*-Value
**Full model**	0.392	15.97	<0.001				
Constant				12.163	5.026	2.420	0.016
Positive relations				0.125	0.049	2.552	0.011
Autonomy				0.062	0.046	1.346	0.180
Environmental mastery				−0.113	0.062	−1.812	0.071
Personal growth				0.233	0.061	3.801	<0.001
Purpose in life				0.034	0.066	0.508	0.612
Self-acceptance				0.004	0.065	0.054	0.957
Negative past				0.009	0.071	0.122	0.903
Hedonistic present				−0.054	0.057	−0.958	0.339
Future				−0.023	0.060	−0.382	0.703
Positive past				0.184	0.073	2.512	0.013
Fatalistic present				−0.196	0.080	−2.437	0.016
**Best predictor model**	0.399	34.9	<0.001				
Constant				13.035	4.013	3.248	0.001
Positive relations				0.137	0.047	2.902	0.004
Environmental mastery				−0.089	0.050	−1.784	0.076
Personal growth				0.248	0.048	5.175	<0.001
Hedonistic present				0.163	0.070	2.320	0.021
Fatalistic present				−0.237	0.068	−3.473	<0.001

**Table 10:** Full and best predictor regression models for *Indifference to Stigma* in Y-Generation sample (N = 217).

“Predictors”	Summary of Model				Coefficients		
	R^2^_adj_	*F*	*p*-Value	Estimate	Std. Error	*t*-Value	*p*-Value
**Full model**	0.361	12.1	<0.001				
Constant				19.789	6.400	3.092	0.002
Positive relations				0.118	0.056	2.125	0.035
Autonomy				0.036	0.049	0.729	0.467
Environmental mastery				−0.044	0.082	−0.530	0.596
Personal growth				0.123	0.069	1.777	0.077
Purpose in life				0.008	0.084	0.091	0.928
Self-acceptance				−0.041	0.068	−0.608	0.544
Negative past				−0.134	0.085	−1.570	0.118
Hedonistic present				0.217	0.065	3.320	0.001
Future				−0.011	0.073	−0.154	0.878
Positive past				0.049	0.087	0.559	0.577
Fatalistic present				−0.404	0.087	−4.632	<0.001
**Best predictor model**	0.374	26.8	<0.001				
Constant				18.559	4.637	4.003	<0.001
Positive relations				0.110	0.051	2.140	0.033
Personal growth				0.110	0.052	2.138	0.034
Negative past				−0.119	0.057	−2.082	0.039
Hedonistic present				0.233	0.062	3.748	<0.001
Fatalistic present				−0.400	0.083	−4.813	<0.001

**Table 11:** Full and best predictor regression models for *Help-seeking Propensity* in a general sample of two generations of participants (N = 473).

Predictors	Summary of Model				Coefficients		
	R^2^_adj_	*F*	*p*-Value	Estimate	Std. Error	*t*-Value	*p*-Value
**Full model**	0.326	21.7	<0.001				
Constant				3.243	3.622	0.895	0.371
Positive relations				0.033	0.034	0.966	0.334
Autonomy				−0.070	0.031	−2.296	0.022
Environmental mastery				0.052	0.046	1.135	0.257
Personal growth				0.170	0.043	4.001	<0.001
Purpose in life				0.037	0.048	0.768	0.443
Self-acceptance				0.025	0.043	0.589	0.556
Negative past				−0.008	0.049	−0.162	0.871
Hedonistic present				0.138	0.039	3.493	<0.001
Future				0.104	0.043	2.432	0.015
Positive past				0.057	0.051	1.119	0.264
Fatalistic present				−0.063	0.054	−1.152	0.250
**Best predictor model**	0.325	38.9	<0.001				
Constant				0.430	2.347	0.183	0.855
Autonomy				−0.055	0.029	−1.902	0.058
Environmental mastery				0.102	0.037	2.734	0.006
Personal growth				0.221	0.034	6.578	<0.001
Hedonistic present				0.115	0.035	3.296	0.001
Future				0.109	0.039	2.812	0.005
Positive past				0.078	0.047	1.652	0.099

**Table 12:** Full and best predictor regression models for *Help-seeking Propensity* in the Z-Generation sample (N = 256).

Predictors	Summary of Model				Coefficients		
	R^2^_adj_	*F*	*p*-Value	Estimate	Std. Error	*t*-Value	*p*-Value
**Full model**	0.300	10.9	<0.001				
Constant				2.601	4.940	0.526	0.599
Positive relations				0.028	0.048	0.571	0.569
Autonomy				−0.051	0.045	−1.135	0.258
Environmental mastery				0.024	0.061	0.398	0.691
Personal growth				0.194	0.060	3.221	0.001
Purpose in life				0.042	0.065	0.640	0.523
Self-acceptance				0.005	0.064	0.084	0.933
Negative past				−0.005	0.069	−0.069	0.945
Hedonistic present				0.084	0.056	1.510	0.132
Future				0.159	0.059	2.706	0.007
Positive past				0.044	0.072	0.608	0.543
Fatalistic present				−0.006	0.079	−0.072	0.942
**Best predictor model**	0.310	39.1	<0.001				
Constant				2.655	2.925	0.908	0.365
Personal growth				0.230	0.038	5.999	<0.001
Hedonistic present				0.081	0.048	1.668	0.097
Future				0.199	0.048	4.120	<0.001

**Table 13:** Full and best predictor regression models for *Help-seeking Propensity* in Y-Generation sample (N = 217).

Predictors	Summary of Model				Coefficients		
	R^2^_adj_	*F*	*p*-Value	Estimate	Std. Error	*t*-Value	*p*-Value
**Full model**	0.343	11.3	<0.001				
Constant				1.477	5.682	0.260	0.795
Positive relations				0.043	0.049	0.864	0.389
Autonomy				−0.082	0.044	−1.877	0.062
Environmental mastery				0.107	0.073	1.472	0.143
Personal growth				0.147	0.061	2.388	0.018
Purpose in life				0.022	0.075	0.299	0.765
Self-acceptance				0.043	0.060	0.720	0.472
Negative past				0.015	0.076	0.198	0.843
Hedonistic present				0.190	0.058	3.271	0.001
Future				0.045	0.065	0.696	0.487
Positive past				0.090	0.077	1.158	0.248
Fatalistic present				−0.129	0.077	−1.667	0.097
**Best predictor model**	0.352	20.5	<0.001				
Constant				3.819	3.980	0.960	0.338
Autonomy				−0.089	0.040	−2.228	0.027
Environmental mastery				0.166	0.054	3.093	0.002
Personal growth				0.191	0.049	3.932	<0.001
Hedonistic present				0.190	0.057	3.365	<0.001
Positive past				0.123	0.068	1.796	0.074
Fatalistic present				−0.137	0.073	−1.879	0.062

**Table 14:** Full and best predictor regression models for *Psychological openness* in a general sample of two generations of participants (N = 473).

Predictors	Summary of Model				Coefficients		
	R^2^_adj_	*F*	*p*-Value	Estimate	Std. Error	*t*-Value	*p*-Value
**Full model**	0.202	11.9	<0.001				
Constant				25.767	3.965	6.499	<0.001
Positive relations				0.068	0.037	1.850	0.065
Autonomy				−0.031	0.033	−0.919	0.358
Environmental mastery				−0.159	0.050	−3.174	0.002
Personal growth				0.174	0.047	3.731	<0.001
Purpose in life				0.044	0.053	0.821	0.412
Self-acceptance				−0.049	0.047	−1.051	0.294
Negative past				−0.070	0.054	−1.296	0.196
Hedonistic present				0.050	0.043	1.166	0.244
Future				0.050	0.047	1.072	0.284
Positive past				0.057	0.056	1.019	0.309
Fatalistic present				−0.253	0.060	−4.242	<0.001
**Best predictor model**	0.200	30.6	<0.001				
Constant				24.159	3.002	8.048	<0.001
Positive relations				0.089	0.035	2.560	0.011
Environmental mastery				−0.132	0.038	−3.463	<0.001
Personal growth				0.194	0.036	5.380	<0.001
Fatalistic present				−0.240	0.050	−4.758	<0.001

**Table 15:** Full and best predictor regression models for *Psychological openness* in the Z-Generation sample (N = 256).

Predictors	Summary of Model				Coefficients		
	R^2^_adj_	*F*	*p*-Value	Estimate	Std. Error	*t*-Value	*p*-Value
**Full model**	0.207	7.07	<0.001				
Constant				23.419	4.995	4.688	<0.001
Positive relations				0.006	0.049	0.118	0.906
Autonomy				0.003	0.046	0.062	0.951
Environmental mastery				−0.124	0.062	−2.006	0.046
Personal growth				0.194	0.061	3.180	0.002
Purpose in life				0.054	0.066	0.825	0.410
Self-acceptance				−0.039	0.065	−0.606	0.545
Negative past				−0.020	0.070	−0.282	0.778
Hedonistic present				−0.069	0.057	−1.219	0.224
Future				0.122	0.059	2.063	0.040
Positive past				0.052	0.073	0.717	0.474
Fatalistic present				−0.178	0.080	−2.233	0.026
**Best predictor model**	0.220	18.96	<0.001				
Constant				22.385	4.132	5.417	<0.001
Environmental mastery				−0.107	0.046	−2.330	0.021
Personal growth				0.186	0.043	4.324	<0.001
Future				0.137	0.052	2.633	0.009
Fatalistic present				−0.221	0.067	−3.288	0.001

**Table 16:** Full and best predictor regression models for *Psychological openness* in the Y-Generation sample (N = 217).

Predictors	Summary of Model				Coefficients		
	R^2^_adj_	*F*	*p*-Value	Estimate	Std. Error	*t*-Value	*p*-Value
**Full model**	0.231	6.9	<0.001				
Constant				24.649	6.520	3.781	<0.001
Positive relations				0.151	0.057	2.669	0.008
Autonomy				−0.029	0.050	−0.577	0.565
Environmental mastery				−0.193	0.084	−2.306	0.022
Personal growth				0.166	0.071	2.353	0.020
Purpose in life				0.066	0.086	0.764	0.446
Self-acceptance				−0.077	0.069	−1.120	0.264
Negative past				−0.094	0.087	−1.079	0.282
Hedonistic present				0.143	0.067	2.136	0.034
Future				−0.038	0.074	−0.517	0.605
Positive past				0.038	0.089	0.430	0.667
Fatalistic present				−0.305	0.089	−3.432	<0.001
**Best predictor model**	0.243	14.9	<0.001				
Constant				18.936	4.515	4.194	<0.001
Positive relations				0.174	0.053	3.319	0.001
Environmental mastery				−0.190	0.061	−3.125	0.002
Personal growth				0.156	0.056	2.776	0.006
Hedonistic present				0.142	0.064	2.227	0.027
Fatalistic present				−0.326	0.083	−3.922	<0.001

## Data Availability

The datasets used and analyzed in the current study are available from the corresponding author upon reasonable request.
